# The Adrenergic Receptor Antagonist Carvedilol Elicits Anti-Tumor Responses in Uveal Melanoma 3D Tumor Spheroids and May Serve as Co-Adjuvant Therapy with Radiation

**DOI:** 10.3390/cancers14133097

**Published:** 2022-06-23

**Authors:** Lina S. Farhoumand, Miltiadis Fiorentzis, Miriam M. Kraemer, Ali Sak, Martin Stuschke, Tienush Rassaf, Ulrike Hendgen-Cotta, Nikolaos E. Bechrakis, Utta Berchner-Pfannschmidt

**Affiliations:** 1 Department of Ophthalmology, University Hospital Essen, University of Duisburg-Essen, 45147 Essen, Germany; lina.farhoumand@stud.uni-due.de (L.S.F.); miltiadis.fiorentzis@uk-essen.de (M.F.); miriam.kraemer@uk-essen.de (M.M.K.); nikolaos.bechrakis@uk-essen.de (N.E.B.); 2 Department of Radiotherapy, West German Cancer Center, University Hospital Essen, University of Duisburg-Essen, 45147 Essen, Germany; ali.sak@uk-essen.de (A.S.); martin.stuschke@uk-essen.de (M.S.); 3 Department of Cardiology and Vascular Medicine, West German Heart and Vascular Center, University Hospital Essen, University of Duisburg-Essen, 45147 Essen, Germany; tienush.rassaf@uk-essen.de (T.R.); ulrike.hendgen-cotta@uk-essen.de (U.H.-C.)

**Keywords:** uveal melanoma, adrenergic receptor blocker, carvedilol, anti-tumor potential, co-adjuvant treatment, radiation

## Abstract

**Simple Summary:**

Decades of research efforts aiming to identify a new therapy for reducing mortality rates in metastatic uveal melanoma (UM) have not been successful. While ß-blockers are already used as the gold standard in other tumors, e.g., infantile haemangiomas, UM has not received much attention. In the present study, we investigated ß-blockers to demonstrate their anti-tumor potential for the treatment of UM. Of the ß-blockers tested, carvedilol was able to block tumor cell viability and the long-term survival of the cells. Considering that brachytherapy is one of the most efficient local therapies for UM, the concurrent treatment of carvedilol and irradiation was performed, which resulted in additive effects. The anti-tumor properties of ß-blockers described in this study could lead to a new co-adjuvant treatment of UM with the aim to reduce the rate of metastasis and thus mortality.

**Abstract:**

Uveal melanoma (UM) is the most common intraocular tumor in adults. Despite local tumor control, no effective therapy has been found to prevent metastasis, resulting in a high mortality rate. In the present study, we evaluated the anti-tumor potential of non-selective ß-blockers in 3D tumor spheroids grown from UM cell lines. Of the various ß-blockers tested, carvedilol and its enantiomers were most potent in decreasing the viability of Mel270 spheroids. Carvedilol at a concentration of 10–50 µM significantly elicited cytotoxicity and induced apoptosis in spheroid cells. In result, carvedilol inhibited tumor spheroid growth and compactness, and furthermore prevented the long-term survival and repopulation of spreading spheroid cells. The drug sensitivity of the different spheroids grown from Mel270, 92-1, UPMD2, or UPMM3 cell lines was dependent on 3D morphology rather than on high-risk cytogenetic profile or adrenergic receptor expression levels. In fact, the monosomy-3-containing UPMM3 cell line was most responsive to carvedilol treatment compared to the other cell lines. The concurrent treatment of UPMM3 spheroids with carvedilol and 5 or 10 Gy irradiation revealed additive cytotoxic effects that provided tumor control. Collectively, our data demonstrate the anti-tumor properties of carvedilol and its enantiomers, which may serve as candidates for the co-adjuvant therapy of UM.

## 1. Introduction

Uveal melanoma (UM) is a tumor comprising neoplastic changes of melanocytes in the choroid, iris, and ciliary body [1]. With an incidence of 5.1 per million, it is known to be the most commonly diagnosed intraocular tumor [2,3]. The risk factors of UM include older age, skin abnormalities, and genetic mutations. The presence of fair skin and eyes, choroidal nevi, and conditions such as oculodermal melanocytosis may lead to a higher incidence [4,5].

There are different genetic aberrations in melanocytes related to UM. Mutations of driver oncogenes of either guanine nucleotide-binding protein Q polypeptide (*GNAQ*) or guanine nucleotide-binding protein alpha-11 (*GNA11*) lead to a dysregulation of G-protein signaling and occur in nearly 80–93% of cases [1]. Even though these genetic mutations play a leading role in regulating the cellular and pathological processes of the disease, they are not known to predict outcome, metastasis risk, or survival [1,6]. Interestingly, this is not the case for genetic monosomy 3. This specific chromosome aberration is linked to many phenotypic changes such as large tumor size or worse intraocular location, and has become the most important prognostic factor [7,8]. The strong correlation between monosomy 3 and higher metastasis risk, and thus poor prognosis, was shown by a study that included 54 patients with UM. The three-year mortality rate of the 30 patients with monosomy 3 was 50%. In contrast, none of the disomy 3 patients developed metastasis and the three-year mortality rate was 0% [8,9]. In addition, losses or mutations of the breast cancer susceptibility gene 1 (*BRCA1*)-associated protein gene (*BAP1*) located on chromosome 3 are associated with a high risk of metastasis and a poor prognosis [10]. The mutation of eukaryotic translation initiation factor 1A, X-linked (*EIF1AX*), has been associated with a lower probability of metastasis in disomy 3 tumors and a good prognosis [6,10].

The primary treatments of the tumor consist of brachytherapy, surgical tumor resection, and, if unavoidable, enucleation of the eye [3]. Episcleral plaque brachytherapy is one of the most effective treatments for UM, preventing many enucleations and increasing the prospect of an eye-preserving outcome [11]. The Collaborative Ocular Melanoma Study (COMS) randomized clinical trial showed no difference in survival between patients treated with brachytherapy and those who received enucleation [12]. Despite good local therapy and control of the tumor, the mortality rate for UM remains high. The 10-year overall survival of patients with primary UM is approximately 50% [13]. A former study examined the cases of 289 consecutive patients with choroidal and ciliary melanoma after radical surgery. The uveal melanoma-related mortality was 31% after 5 years and 49% after 25 years. The verified cause of death was confirmed in 128 patients, of which 63% were due to melanoma metastasis [14]. There have been many attempts to find new treatments for metastatic control in UM. Apart from a recent study of tebentafusp, no therapy has been found to improve overall survival in metastatic tumors [15,16]. Although UM and cutaneous melanoma share the same origin from melanocytes, UM differs markedly from cutaneous melanoma in many factors, and the therapeutic successes of cutaneous melanoma are not shared [13].

For decades, ß-adrenoceptor blockers have been used to treat cardiovascular diseases such as high blood pressure [17]. After various studies proved that stress and thus the fight-or-flight neurotransmitters adrenaline and noradrenaline trigger cancer development, the field of application for ß-adrenergic receptor antagonists has changed [18]. While the anti-tumor effects are still under investigation in various studies, they have already become the first therapeutic choice for infantile hemangiomas [19]. The effect of ß receptors on tumor progression included cell proliferation and vascular events, resulting not only in cancer cell invasion but also in metastasis [18,20]. The upregulated expression of ß-adrenergic receptors in cutaneous melanoma and the anti-tumor properties of the non-selective ß-blocker propranolol have been demonstrated in many studies [21,22,23,24]. Importantly, a prospective study using propranolol as an off-label therapy revealed an 80% reduction in the risk of melanoma recurrence [25]. Recently, a study demonstrated the presence of ß-adrenoceptors in UM and the anti-tumor effects of ß-blocker propranolol. The non-selective ß-blocker propranolol reduced proliferation and attenuated the migration of UM cells, among other effects [23].

In the present study, we aim to identify the most effective anti-tumor ß-blocker for the treatment of UM and perform a screening with various ß-blockers. To better represent the architecture and cellular arrangement of UM tumors, 3D tumor spheroid models are used. Spheroids are particularly well suited for testing the effect of drugs, as they exhibit a lower penetration for therapeutic molecules [26,27,28]. In our study, we examine the anti-tumor responses of spheroids generated from four UM cell lines with different cytogenetic risk profiles to represent the heterogeneity of UM. Of the ß-blockers tested, carvedilol and its R-enantiomer are found to be most effective in inhibiting spheroid cell viability. In order to evaluate the efficacy of carvedilol, spheroid growth and viability are assessed, in addition to cytotoxicity, apoptosis, and long-term repopulation capacity. Finally, the concurrent treatment of carvedilol with radiation is investigated to determine whether carvedilol can serve as a candidate for the co-adjuvant therapy of UM.

## 2. Materials and Methods

### 2.1. Culture of Cell Lines

The UM cell line Mel270 was kindly provided by Dr. K. Griewank (Department of Dermatology, University Hospital Essen, Essen, Germany). Mel270 originated from a large recurrent tumor after prior irradiation [7]. Uveal melanoma primary cell lines UPMD2 and UPMM3 and UM cell line 92-1 were kindly provided by Dr. M. Zeschnigk (Institute of Human Genetics, University Hospital Essen, Essen, Germany). The 92-1 cell line was derived from a large primary tumor [7]. UPMD2 and UPMM3 were derived from untreated UM, characterized regarding chromosome 3 status, and provided in low passages [29]. All cell lines were characterized previously and the cytogenetic and morphologic characteristics are summarized in Table 1. All cell lines were authenticated by short tandem repeat profiling according to published data.

Mel270 and 92-1 cell lines were maintained in RPMI 1640 medium (GIBCO, Fisher Scientific, Thermo Fisher Scientific Inc., Waltham, MA, USA), and UPMD2 and UPMM3 in Hams/F12 medium (PAN-Biotech GmbH, Aidenbach, Germany). Medium was supplemented with 10% fetal calf serum (Sigma-Aldrich, St. Luois, MO, USA/Chemie GmbH, Steinheim, GE) and 1% penicillin–streptomycin (5000 U/mL, PAN BIOTECH, Aidenbach, GE). Primary human left ventricular cardiomyocytes isolated from an adult donor were purchased (HCM, PromoCell, Heidelberg, Germany) and maintained in myocyte growth medium (PromoCell, Heidelberg, Germany). Medium was refreshed two times per week. The cell lines were incubated in a humidified incubator (37 °C, 5% CO_2_) for the indicated period of time.

### 2.2. Generation of 3D Tumor Spheroids

Spheroids were generated in round-bottom 96-well ultra-low attachment plates (PHC Corporation, Tokyo, Japan) by seeding 5 × 10^3^ living cells in 100 µL of the cell culture medium per well. In order to generate uniform and compact spheroids, the spheroids were cultured for 7 days while the medium was refreshed once, as described earlier [32]. The spheroid cultures were maintained in a humidified incubator (37 °C, 5% CO_2_) for the indicated period of time.

### 2.3. Drug Treatment

Non-selective ß-blockers pindolol (CAS No.: 13523-86-9), timolol maleate salt (CAS No.: 26921-17-5), sotalol hydrochloride (CAS No.: 959-24-0), propranolol hydrochloride (CAS No.: 318-98-8), labetalol hydrochloride (CAS No.: 32780-64-6), and carvedilol (CAS No.: 72956-09-3) were purchased from Sigma-Aldrich (St. Louis, USA/Chemie GmbH, Steinheim, GE). In other experiments, R-(+)-carvedilol (CAS No.: 95093-99-5), S-(−)-carvedilol (CAS No.: 95094-00-1), and carvedilol (equimolar racemate of R-(+)-and S-(−)-carvedilol, CAS No.: 72956-09-3) were purchased from Toronto Research Chemicals (Ontario, Canada). A stock solution of 50 mM in dimethyl sulfoxide (DMSO) was prepared. The spheroids or the cells were incubated in the respective medium supplemented with various concentrations of ß-blockers for the indicated period of time. Control spheroids or cells (0 µM ß-blocker) received DMSO in the same concentration as present in the highest concentration of the blocker in the respective assays. DMSO did not affect the viability of the spheroids at any concentration tested equivalent to 200 µM ß-blocker.

### 2.4. Radiation Treatment

An X-ray irradiator RS320 (Xstrahl Ltd., Surry, UK) was used to irradiate the spheroid cultures at 300 kV, 10 mA, and a dose rate of 0.9 Gy/min. Irradiation was performed in 100 µL medium with or without carvedilol in multi-wells with 5 or 10 Gy within one hour.

### 2.5. Determination of Spheroid Size and Compactness

The imaging of spheroid cultures (*n* = 5 each condition) was conducted on day 7 and day 14 using a Zeiss Primovert bright-field microscope at 4× magnification. Zeiss Axiocam 105 and ZENcore software were used to capture the images and images were then analyzed using image processing software ImageJ Fiji (MPI-CBG, Dresden, Germany). Spheroid size and compactness were determined by calculating the cross-sectional area of spheroids (µm^2^) and by calculating the optical density of the spheroid area (mean grey value). The cross-sectional area or density of the treated spheroids was normalized to the mean of the untreated spheroids before treatment and is given in arbitrary units (AU).

### 2.6. Spheroid Viability Assay

Tumor spheroid viability (*n* = 8 each condition) was determined using the CellTiter-Glo 3D Cell Viability assay (Promega GmbH, Walldorf, Germany) to measure the ATP content of the assessed spheroids. To allow the complete lysis of spheroid cells and the release of ATP, equal amounts of spheroid cultures and reagent were mixed by pipetting up and down for 30 s. The mixture was transferred to white opaque-walled multi-well plates (Nunc, Thermo Fisher Scientific, Roskilde, Denmark). The resulting luminescence was recorded with a FluostarOmega reader (BMG LABTECH, Ortenberg, Germany) after five minutes of incubation on a shaker at 750 rpm and an additional 25 min of incubation, to stabilize luminescence signal. The ATP luminescence (relative light units) of the treated spheroids was normalized to the ATP luminescence of the control spheroids and is given in arbitrary units (AU).

### 2.7. Spheroid Cytotoxicity Assay

The cytotoxicity of carvedilol for spheroids (*n* = 8 each condition) was assessed by measuring the release of lactate dehydrogenase (LDH) from spheroids into the cell culture medium using the LDH-Glo cytotoxicity assay (Promega GmbH, Walldorf, Germany). The supernatant of spheroid cultures was collected, diluted 1:20 in storage buffer (200 mM Tris/HCl, pH 7.3; 10% Glycerol, 1% BSA), and stored at −20 °C. Equal amounts of spheroid supernatant (final concentration 1:100) and LDH-detection reagent were mixed in white opaque-walled multi-well plates (Nunc, Thermo Fisher Scientific, Roskilde, Denmark). After 60 min of incubation, the luminescence was recorded using a reader FluostarOmega (BMG LABTECH, Ortenberg, Germany). The LDH luminescence (relative light units) of the treated spheroids was normalized to the LDH luminescence of the control spheroids and is given in arbitrary units (AU).

### 2.8. Spheroid Apoptosis Assay

Spheroid cell apoptosis (*n* = 8 spheroids each condition) was assessed by detecting caspase 3/7 activity in spheroids using the Caspase-Glo 3/7 assay (Promega GmbH, Walldorf, Germany). Equal amounts of spheroid cultures and substrate solution were mixed by pipetting up and down for 30 s to enable the complete lysis of the spheroid cells. The mixture was transferred to a white opaque-walled multi-well plate (Nunc, Thermo Fisher Scientific, Roskilde, Denmark). After 30 s on a shaker at 500 rpm and a further 40 min of incubation, the luminescence was recorded using a reader FluostarOmega (BMG LABTECH, Ortenberg, Germany). The caspase 3/7 luminescence (relative light units) of the treated spheroids was normalized to the caspase 3/7 luminescence control spheroids and is given in arbitrary units (AU).

### 2.9. Spheroid Cell Survival Assay

Four days after treatment, spheroid cultures (*n* = 12 each condition) were individually transferred to flat bottom 24-well plate dishes (Cellstar, Greiner Bio-One GmbH, Frickenhausen, Germany). Spheroid cells were allowed to attach to the uncoated flat plastic bottom to enable cell out-growth and repopulation. Cells were cultured until the control cells were 90% confluent. Medium was refreshed at least once per week. Finally, adherent cells were fixed with 4% formaldehyde for 5 min, and cell nuclei/DNA were stained with 0.05% crystal violet (CV) for 15 min and washed 3× with water. The absorbance of CV was measured at OD 540 nm using a reader ClarioStar Plus (BMG LABTECH, Ortenberg, Germany). The CV absorbance (relative absorbance units) of the treated spheroids was normalized to the CV absorbance of the control cultures and is given in arbitrary units (AU). The CV-stained cultures were imaged using a Zeiss Primovert bright-field microscope at 4× magnification. Images were recorded with Zeiss Axiocam 105 and ZENcore software.

### 2.10. Cell Viability Assay

For cell culture experiments 5 × 10^3^ living cells were seeded in flat-bottom 96-well plates (Sarstedt, Nürnbrecht, Germany) in 100 µL of the cell culture medium overnight. Cell cultures were treated with carvedilol (*n* = 8 each condition) and incubated for 7 days. Cell viability was assessed using the CellTiter-Glo 2D viability assay (Promega GmbH, Walldorf, Germany). To enable the complete lysis of the cells, a volume of CellTiter-Glo reagent equal to the volume of cell culture medium present in each well was added and mixed by pipetting up and down for 10 s. The mixture was transferred to white opaque-walled multi-well plates (Nunc, Thermo Fisher Scientific, Roskilde, Denmark) and incubated for two minutes on a shaker at 750 rpm and a further 10 min under light protection. The luminescence of the samples was recorded using a reader FluostarOmega (BMG LABTECH, Ortenberg, Germany). The ATP luminescence (relative light units) of the treated spheroids was normalized to the ATP luminescence of the control cells and is given in arbitrary units (AU).

### 2.11. Immunofluorescence Microscopy

For the detection of adrenergic receptors, 10^5^ cells were grown on coverslips placed in 24-well plates. Cells were fixed with 4% formaldehyde for 10 min and permeabilized with 0.1%Nonidet P40 for 2 min. The samples were washed with PBS 3 times for 5 min each. The cells were blocked with 3%BSA/PBS for 30 min and incubated with primary antibodies rabbit anti-ADRA1A (RRID:AB_10857196), ADRB1 (RRID: AB_10885544), ADRB2 (1:50, RRID: AB_10855871, Bios antibodies, Biozol Diagnostics Vertrieb, Eching, Germany), or rabbit anti-MLANA (1: 100, RRID: AB_2799664, Cell Signaling, Leiden, The Netherlands) for 1 h. After the samples were washed with PBS 3 times for 5 min each, the cells were incubated with secondary antibody Alexa fluor 594 goat anti-rabbit antibody (1:400, Invitrogen, RRID: AB_2534095, Thermo Fischer Scientific Inc., Waltham, MA, USA) for 1 h. Cell nuclei were stained with DAPI (1:10.000, Invitrogen, Thermo Fischer Scientific Inc., Waltham, MA, USA) and washed with PBS 3 times for 5 min each. Specimens were embedded with ProLong Gold Antifade Mountant (Invitrogen, Thermo Fischer Scientific Inc., Waltham, MA, USA). Immune-stained cells were imaged using an Olympus BX51 epifluorescence microscope at 60× magnification. Images were recorded with Olympus DP70 1.5 Megapixel color ccd camera.

### 2.12. Statistical Analysis

Statistical analyses of the data were performed using GraphPad Prism (GraphPad Prism 8.4.3 software, GraphPad Software Inc., San Diego, CA, USA). Data were analyzed by one-way ANOVA or two-way ANOVA and Tukey’s multiple comparisons test and considered statistically significant at a value of *p* < 0.05. The significance levels indicated are as follows: * *p* < 0.05, ** *p* < 0.01, *** *p* < 0.001, **** *p* < 0.0001.

## 3. Results

### 3.1. Identification of Carvedilol as the Most Potent Anti-Tumor ß-Blocker

**Figure 1:**
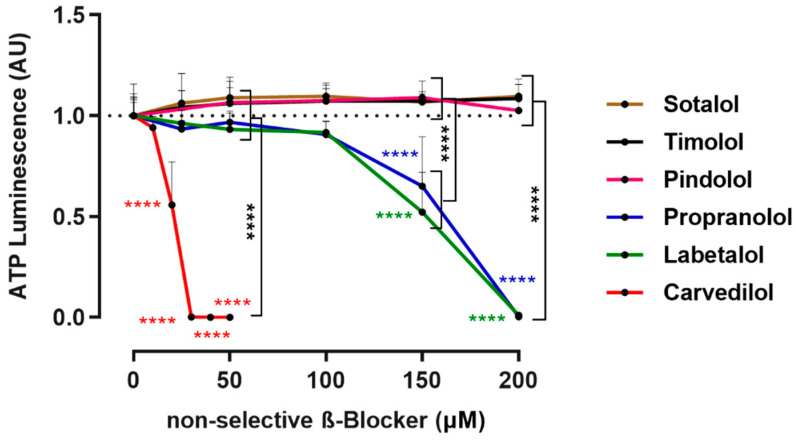
Screening of non-selective ß-blockers for anti-tumor properties in 3D tumor spheroids. Spheroids were generated from a Mel270 cell line and treated with the respective non-selective ß-blocker at the indicated concentration. After 7 days of incubation, spheroid viability was assayed with an ATP luminescence assay. The ATP luminescence of the treated spheroids was normalized to the ATP luminescence of the control spheroids (0 µM ß-blocker) and is given in arbitrary units (AU). Representative results from at least 3 independent experiments for each ß-blocker are shown. The means +/− SD of *n* = 8 spheroids for each condition are shown. Statistical analysis by two-way ANOVA and Tukey’s multiple comparisons test, and the significance levels of the treated spheroids in relation to the control spheroids (0 µM ß-blocker) are displayed in color. Significance levels are indicated at **** *p* < 0.0001.

Frequently used non-selective ß-blockers were screened for their anti-tumor potential in 3D tumor spheroids derived from cell line Mel270 by assessing spheroid viability (Figure 1). Mel270 cell lines are derived from recurrent tumors after prior irradiation and form relatively large and uniform spheroids, as shown previously [32]. Therefore, Mel270 spheroids were chosen for the valid 3D cell culture model for therapy-resistant UM tumors. The ß-blockers sotalol, timolol, and pindolol did not have any effect on spheroid viability. In contrast, ≥150 µM propranolol or labetalol and ≥20 µM carvedilol decreased spheroid viability in a concentration-dependent manner (Figure 1). Interestingly, the ß-blockers labetalol and carvedilol are known to have an α-blocking activity as well. Of the tested α/ß-blockers, carvedilol was most potent in decreasing spheroid viability (Figure 1).

**Figure 2:**
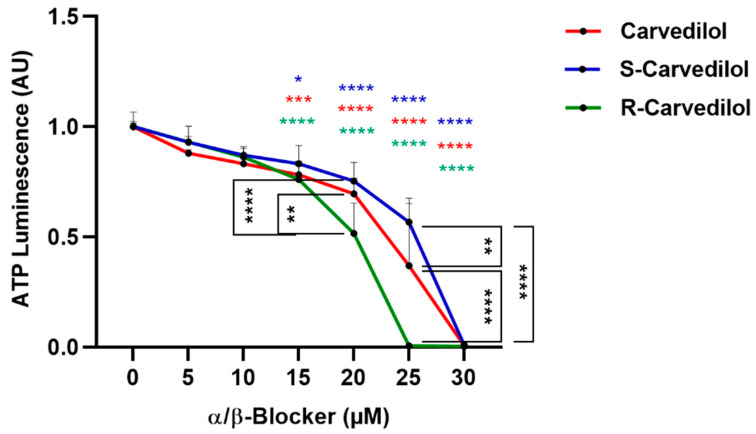
Comparison of cytotoxic effects of carvedilol, S- and R-carvedilol on 3D tumor spheroid viability. Mel270 spheroids were treated with either carvedilol or enantiomers S-carvedilol (α/ß-blocker) and R-carvedilol (α-blocker) at the indicated concentrations. After 7 days of incubation, spheroid viability was assayed with an ATP luminescence assay. The ATP luminescence of the treated spheroids was normalized to the ATP luminescence of the control spheroids (0 µM ß-blocker) and is given in arbitrary units (AU). Representative results from 3 independent experiments are shown. The means ± SD of *n* = 8 spheroids for each condition are shown. Statistical analysis by two-way ANOVA and Tukey’s multiple comparisons test are shown with significance levels indicated at * *p* < 0.05, ** *p* < 0.01, *** *p* < 0.001, and **** *p* < 0.0001. Significance levels of the treated spheroids in relation to the control spheroids (0 µM ß-blocker) are displayed in color.

However, the clinically used carvedilol is a racemic mixture consisting of equal amounts of S- and R-enantiomers which have different blocking activities. S-carvedilol is known to have ß1/2- and α1-blocking activity, while R-enantiomer is solely α1-blocking [33,34]. All types of carvedilol significantly inhibited the viability of Mel270 spheroids at a concentration range of 15–30 µM. Of note, the non-ß-blocking R-carvedilol reduced viability most efficiently while S-carvedilol was less efficient, implicating that α1-blocking is involved in reducing spheroid viability (Figure 2).

### 3.2. Anti-Tumor Responses of 3D Tumor Spheroids Treated with Carvedilol

**Figure 3:**
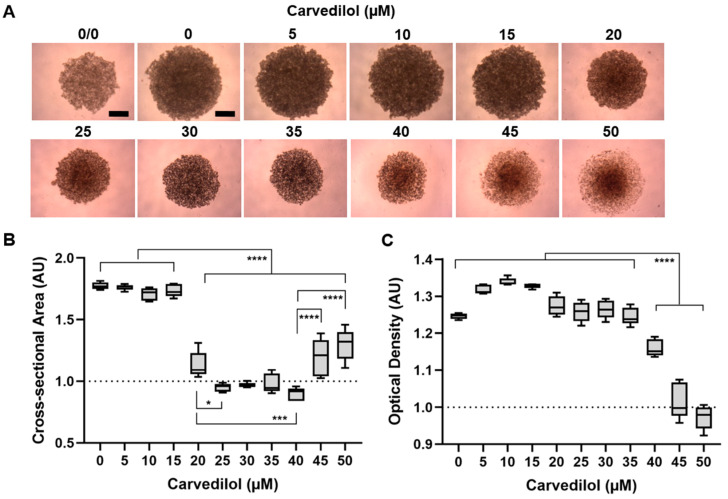
Effects of carvedilol on 3D tumor spheroid growth and compactness. Mel270 spheroids were treated with carvedilol at the indicated concentrations for 7 days. The spheroids were microscopically imaged and size and density were measured. (**A**) Representative microscopic images of the spheroids are shown. Untreated spheroids just before treatment (0/0), and spheroids after 7 days of incubation with 0–50 µM carvedilol (4× magnification): scale bars indicate 500 µm. (**B**,**C**) Spheroid cross-sectional area and optical density were determined and normalized to spheroids before treatment (0/0). Box plots with min to max whiskers and a median of *n* = 5 spheroids for each condition are shown. The mean of the cross-sectional area or the optical density of the spheroids before treatment (0/0) are indicated by dotted lines. Representative results from 3 independent experiments are shown. Statistical analysis by one-way ANOVA and Tukey’s multiple comparisons test, significance levels are indicated at * *p* < 0.05, *** *p* < 0.001, and **** *p* < 0.0001.

**Figure 4:**
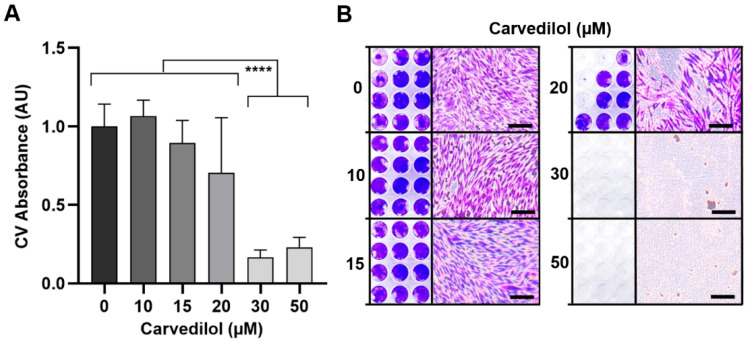
Long-term survival and repopulation of carvedilol-treated 3D tumor-spheroid cells. Spheroids were generated from a Mel270 cell line and treated with carvedilol at the indicated concentrations. After four days, the spheroid cultures were individually transferred to flat-bottom wells and cultured until control cells (0 µM carvedilol) were confluent. After 2 weeks of culture, the cells were stained crystal violet (CV). (**A**) Measurements of absorption of CV-stained cells. The CV absorption of the treated cells was normalized to the CV absorption of the control cells (0 µM carvedilol) and is given in arbitrary units (AU). The means ± SD of *n* = 12 spheroids for each condition are shown. Representative results from 3 independent experiments are shown. Statistical analysis was performed with one-way ANOVA and Tukey’s multiple comparisons test and significance levels are indicated at **** *p* < 0.0001. (**B**) Representative images of CV staining of the spheroid-derived cell cultures (*n* = 12) and microscopic images of cells (10× magnification) for each condition are shown. The scale bars indicate 100 µm.

**Figure 5:**
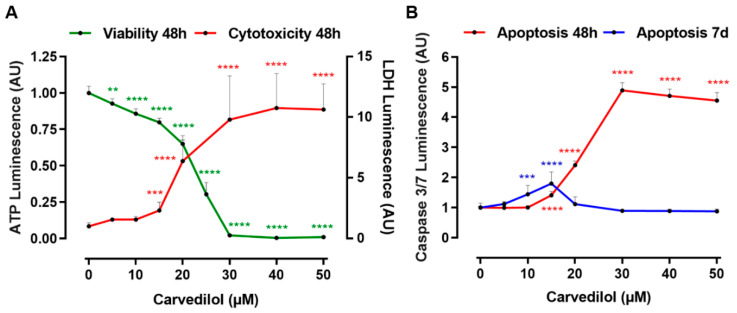
Cytotoxicity of carvedilol and the induction of apoptosis in 3D tumor spheroids. Spheroids were generated from a Mel270 cell line. (**A**) Spheroids were treated with carvedilol at the indicated concentration for 48 h. Spheroid viability was assayed with an ATP luminescence assay. The ATP luminescence of the treated spheroids was normalized to the ATP luminescence of the control spheroids (0 µM carvedilol) and is given in arbitrary units (AU). Representative results from 3 independent experiments are shown. The means SD of *n* = 8 spheroids for each condition are shown. The cytotoxicity of carvedilol was assayed with the LDH-Glo cytotoxicity assay. The LDH luminescence was normalized to the LDH luminescence of the control spheroids (0 µM carvedilol) and is given in arbitrary units (AU). The means ± SD of *n* = 8 spheroids for each condition are shown. (**B**) Spheroids were treated with carvedilol at the indicated concentration for either 48 h or 7 days. Spheroid apoptosis was assayed with a caspase 3/7 luminescence assay. The caspase 3/7 luminescence of the treated spheroids was normalized to the luminescence of the control spheroids (0 µM Carvedilol) and is given in arbitrary units (AU). Representative results from 3 independent experiments are shown. The means ± SD of *n* = 8 spheroids for each condition are shown. Statistical analysis was performed with one-way ANOVA and Tukey’s multiple comparisons test, and significance levels are indicated at ** *p* < 0.01, *** *p* < 0.001, and **** *p* < 0.0001.

We next aimed to analyze the anti-tumor responses of the Mel270 spheroids treated with carvedilol in more detail (Figure 3, Figure 4 and Figure 5). Since the racemic mixture of carvedilol is the clinically available form, it was used in further experiments. The microscopic examination of treated spheroids revealed that carvedilol dose-dependently changed spheroid size and appearance (Figure 3). Drug concentrations ≥20 µM caused a significant decrease in the spheroid cross-sectional area indicating the inhibition of spheroid growth (Figure 3A,B). However, at higher concentrations of carvedilol ≥45 µM, the area of the spheroids appeared to increase again (Figure 3A,B). Furthermore, spheroid density was significantly decreased at concentrations ≥40 µM, indicating lower spheroid compactness. Microscopic observation revealed that these spheroids lost cohesion, and at ≥45 µM carvedilol the outer layers of the spheroids disintegrated into large loose cell aggregates (Figure 3A,C). These data suggest that carvedilol ≥20 µM blocks spheroid cell proliferation and higher doses ≥40 µM result in the cell dissociation of remaining spheroids.

In order to determine the tumor control potential, we investigated the long-term cell survival and repopulation capacity of carvedilol-treated Mel270 spheroids. Therefore, each spheroid culture was individually seeded in a flat-bottom well to allow cell out-growth and was cultured until control cells were confluent (Figure 4). Carvedilol concentrations ≥ 30 µM completely prevented the survival and repopulation of spreading spheroid cells (Figure 4A). Microscopic examination of the stained cell cultures confirmed that no cells remained after treatment with carvedilol ≥ 30 µM, but brownish cell debris did (Figure 4B). Even a lower dose of 20 µM carvedilol reduced the long-term survival of spheroid cells in some cultures (Figure 4B). These data suggest that a carvedilol concentration of ≥30 µM allows long-term tumor control.

Furthermore, we analyzed the early effects of carvedilol on Mel270 spheroids (Figure 5). Following a 48 h exposure, spheroid viability was decreased in a concentration-dependent manner starting at 5 µM, and was totally blocked at ≥30 µM of the drug. Consistent with this finding, carvedilol was increasingly cytotoxic, reaching a maximum at ≥30 µM (Figure 5A). The increasing release of LDH from the spheroid cells measured by the cytotoxicity assay indicated that necrosis-like processes were induced in spheroids in response to ≥15 µM carvedilol. Moreover, measurements of caspase 3/7 activity in spheroids revealed that carvedilol induced apoptotic pathways in spheroids from 15 µM to a maximum of 30 µM after 48 h of incubation time. Even lower carvedilol concentrations of 10–15 µM significantly induced apoptosis after a prolonged incubation period of 7 days (Figure 5B).

The results suggest that the anti-tumor activity of carvedilol involves an early induction of apoptosis and necrosis-like processes, resulting in the inhibition of spheroid growth and compactness. More importantly, carvedilol blocks long-term survival and the repopulation capacity of cells spreading from treated spheroids.

### 3.3. Comparison of Anti-Tumor Responses of Various 3D Tumor Spheroids and 2D Cell Lines

**Figure 6:**
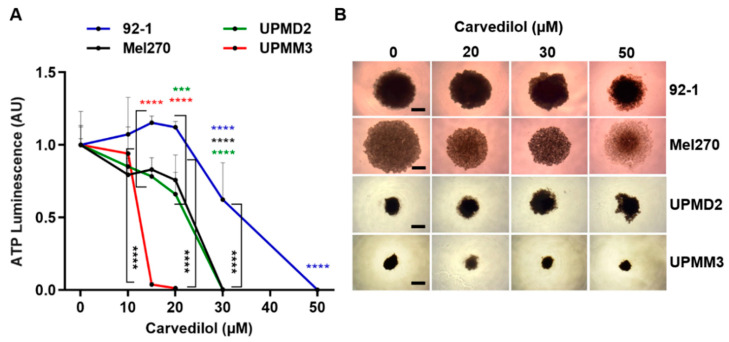
Cytotoxic effects of carvedilol on the viability of various 3D tumor spheroid models. Spheroids were generated from cell lines 92-1 and Mel270 or primary cell lines UPMD2 and UPMM3. Spheroids were treated with carvedilol at the indicated concentration for 7 days. (**A**) Spheroid viability was assayed with an ATP luminescence assay. The ATP luminescence of the treated spheroids was normalized to the ATP luminescence of the control spheroids (0 µM carvedilol) and is given in arbitrary units (AU). Representative results from 3 independent experiments are shown. The means ± SD of *n* = 8 spheroids for each condition are shown. Statistical analysis was performed with two-way ANOVA and Tukey’s multiple comparisons test, and the significance levels of the treated spheroids in relation to the control spheroids (0 µM ß-blocker) are indicated in color. Significance levels are indicated at *** *p* < 0.001, **** *p* < 0.0001. (**B**) Representative microscope images (4× magnification) of 3D tumor spheroids at the indicated concentrations of carvedilol on day 7 are shown. Scale bars indicate 500 µm.

UM cell lines are known to differ genetically as well as in cell morphology and in proliferation rate, which may reflect the inter- or intra-patient heterogeneity of UM (Table 1). To account for the heterogeneity of UM, we additionally generated 3D tumor spheroids from the two primary cell lines UPMD2 and UPMM3, and another established cell line 92-1 for comparison with the Mel270 spheroids (Table 1). The various spheroid types were differently affected by carvedilol treatment (Figure 6). Among the spheroid types, the 92-1 spheroids turned out to be less responsive to carvedilol treatment. The 92-1 spheroid viability was reduced in response to 30 to 50 µM carvedilol, whereas the viability of the UPMD2 spheroids and Mel270 spheroids was already blocked at 30 µM carvedilol. Of note, the monosomy-3-containing UPMM3 spheroids were most responsible, and viability was blocked already with 15–20 µM carvedilol (Figure 6A).

However, the spheroids differed in size and compactness (Figure 6B). Cell lines 92-1 and Mel270 generated much larger spheroids than the primary cell lines UPMD2 and UPMM3. UPMM3 spheroids were the smallest, while Mel270 spheroids were less compact, which is consistent with our earlier findings [32]. Despite these morphological differences, the microscopic examination revealed that carvedilol treatment led to decreased size and/or the disaggregation of spheroids in a range of 20–50 µM (Figure 6B).

**Figure 7:**
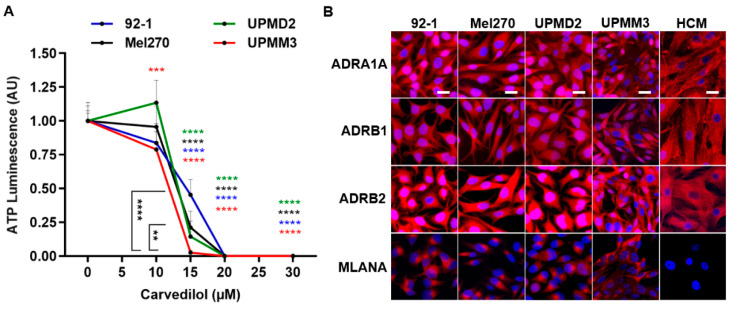
Effects of carvedilol on various uveal melanoma cell lines and the expression of adrenergic receptors. (**A**) Uveal melanoma cell cultures were treated with carvedilol at the indicated concentration for 7 days. Cell viability was assayed with an ATP luminescence assay. The ATP luminescence of the treated cells was normalized to the ATP luminescence of the control cells (0 µM carvedilol) and is given in arbitrary units (AU). Representative results from 3 independent experiments are shown. The means ± SD of *n* = 8 cell cultures for each condition are shown. Statistical analysis was performed with two-way ANOVA and Tukey’s multiple comparisons test, and the significance levels of the treated cells in relation to the control cells are indicated in color. Significance levels are indicated at ** *p* < 0.01, *** *p* < 0.001, and **** *p* < 0.0001. (**B**) Uveal melanoma cell lines or human cardiomyocytes (HCM) grown on coverslips and adrenergic receptors α1 (ADRA1A), ß1 (ADRB1) and ß2 (ADRB2) or melanoma marker MLANA were detected by immunofluorescence (displayed in red colour). Human cardiomyocytes served as positive controls for the expression of adrenergic receptors. Cell nuclei were counterstained with DAPI (displayed in blue). Merged images are shown, and the scale bars indicate 20 µm.

In addition, we analyzed the viability of the respective cell lines in response to carvedilol treatment to determine whether the different sensitivities of the spheroids were due to the different 3D morphologies or whether cell-type immanent factors were involved (Figure 7). The viability of all cell lines was reduced dose-dependently in the concentration range of 15–20 µM carvedilol in a comparable manner. Carvedilol sensitivity was significantly higher in UPMM3 cells when compared to the other cell lines (Figure 7A). We next examined whether the carvedilol sensitivity of UPMM3 cells was related to altered adrenergic receptor levels. The adrenergic receptors A1A, B1, and B2 were consistently expressed across all cell lines tested. In general, the UM cell lines expressed higher levels of B2 compared to the B1 or A1A receptors (Figure 7B).

Interestingly, the drug sensitivity of the cell lines 92-1, Mel270, and UPMD2 was higher compared to their respective spheroids. However, the drug sensitivity of the cell line UPMM3 and the derived small spheroids was comparable (Figure 6A and Figure 7A). The data indicate that the potency of carvedilol was mainly affected by the 3D morphology of the spheroids rather than by cell-type characteristics such as morphology, doubling time, or genetic profile. However, adrenergic receptor levels may also play a role in the drug responsiveness of the tumor cells.

### 3.4. Combined Treatment of Carvedilol and Radiation

**Figure 8:**
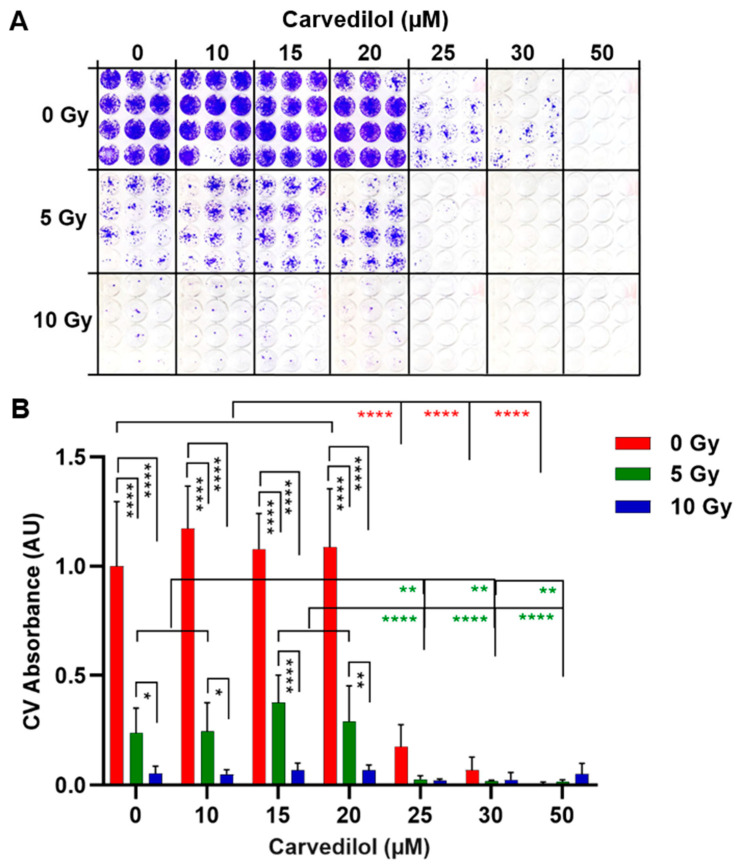
Long-term survival and repopulation after co-treatment of 3D tumor spheroids with carvedilol and radiation. Spheroids were generated from the UPMM3 cell line and treated with carvedilol and/or radiation at 5 or 10 Gy. After four days of incubation, individual spheroids were transferred into flat-bottom wells to enable the out-growth of cells. Cells were cultured until control cells (0 µM carvedilol, 0 Gy) were confluent. After 4 weeks, cell cultures were stained crystal violet (CV). (**A**) Representative images of CV-stained cell cultures (*n* = 12) for each condition are shown. (**B**) Measurements of CV absorption (*n* = 12 each condition). The CV absorption of the treated cells was normalized to the CV absorption of the control cells (0 µM Carvedilol, 0 Gy) and is given in arbitrary units (AU). Representative results of 3 independent results are shown. The means ± SD of *n* = 12 spheroids for each condition are shown. Statistical analysis was performed with two-way ANOVA and Tukey’s multiple comparisons test, and significance levels are indicated at * *p* < 0.05, ** *p* < 0.01, **** *p* < 0.0001.

To investigate whether carvedilol could be a novel therapeutic option for UM with a high-risk status for metastasis and worse prognosis, we evaluated the tumor-control potential of carvedilol for spheroids derived from the UPMM3 cell line. The UPMM3 cell line originated from an untreated primary tumor with monosomy 3 and thus exhibited the highest risk status for metastasis [7,8,9,29]. Following the idea that ß-blockers can be used clinically as co-adjuvant therapy to delay the recurrence or metastasis of UM, UPMM3 spheroids were irradiated and additionally treated with carvedilol. Thereafter, treated spheroids were individually transferred to long-term cultures until control cultures reached confluence (Figure 8). Carvedilol ≥25 µM significantly reduced long-term survival and at 50 µM completely prevented the repopulation of spreading spheroid cells (Figure 8A,B). The irradiation of spheroids at 5 Gy or 10 Gy significantly reduced long-term survival in a dose-dependent manner, but did not prevent the repopulation of the tumor cells. In contrast, the irradiation of spheroids with 5 or 10 Gy combined with carvedilol ≥25 µM completely blocked the repopulation of the spreading spheroid cells. Of note, a combination of a low dose of 5 Gy with 25 µM carvedilol was even more effective than each treatment on its own (Figure 8A,B). The results indicated that carvedilol can be co-administered with radiation without compromising the efficacy of either carvedilol or radiation. Moreover, lower doses of both carvedilol and radiation in combination allowed tumor control of the spheroids derived from the UM cell line with high-risk status.

## 4. Discussion

The present study investigated the effect of non-selective ß-blockers in UM 3D spheroid culture, and additionally evaluated ß-blockers as a co-adjuvant treatment to radiation therapy. Although the anti-tumor properties of ß-adrenergic receptor antagonists have already been demonstrated in multiple in vitro studies of other tumor types [35], this is the first study screening various non-specific ß-blockers for anti-tumor potential in UM cells and to further examine 3D tumor spheroids grown from UM cell lines with different genotypes and risk-status for metastasis.

In our study, the superior effect of the non-selective ß-blockers of carvedilol, labetalol, and propranolol was observed in large 3D spheroids derived from a Mel270 cell line. The most potent blocker, carvedilol, was further investigated and morphological changes in 3D spheroids, as well as apoptotic and necrotic effects, were observed. Regarding the inhibition of viability or the induction of apoptosis and necrosis-like processes, 50% of the maximal effects were observed at a carvedilol dose of ~20 µM. Carvedilol doses >20 µM resulted in the inhibition of spheroid growth and the blockage of long-term survival and the repopulation of tumor cells. Most importantly, the inhibition of the viability of 3D spheroids derived from Mel270 was detected and confirmed in spheroids of cell lines 92-1, UPMD2, and UPMM3 in a concentration range of 15–50 µM. In 2D cultures, even lower doses of carvedilol were sufficient to block viability compared to the larger and more compact spheroids.

Mel270 and UPMM3 spheroids were used for further investigations, based on their characteristics. Mel270 originated from a recurrent tumor after prior irradiation and thus escaped radiation therapy [7]. Previous studies have confirmed that Mel270 spheroids are less responsive to radiation and electrochemotherapy with bleomycin compared to other UM cell lines 92-1, UPMD2, or UPMM3 [32,36]. However, in the present study, the tumor control of Mel270 spheroids was achieved by carvedilol treatment. Furthermore, we confirmed the tumor control potential of carvedilol in 3D spheroids grown from the UPMM3 cell line. The UPMM3 cell line originated from an untreated primary tumor. Among the cell lines, only UPMM3 contained monosomy 3 and thus exhibited a high-risk status for metastasis [29]. The long-term survival assay of UPMM3 spheroids revealed that carvedilol combined with irradiation inhibited out-growth and the repopulation of tumor cells more effectively than each treatment alone. The inhibition of tumor cell out-growth and repopulation would delay or, at best, prevent recurrence and metastasis.

ß-adrenoceptors can be divided into three generations according to their pharmacological properties. First-generation non-selective ß-blockers, such as sotalol, timolol, pindolol, and propranolol, act on both ß1 and ß2 receptors. In contrast, second-generation ß-blockers are ß1-selective, and third-generation blockers, such as carvedilol or labetalol, have an additional blocking effect on α1 receptors [17,35,37]. Furthermore, the structures of the various ß-blockers exhibited differences in the number of chiral centers and the composition of the R- and S-enantiomers in racemic mixtures [38]. Carvedilol contains one chiral center that forms a racemic mixture in which the S-stereoisomer exhibits ß1 and α1 receptor antagonism, whereas the R-stereoisomer solely blocks the α1 receptor [33,34]. These different receptor affinities of ß receptor blockers and the composition of racemic mixtures complicate the determination of their anti-tumor effects.

In our study, the superior effects of carvedilol, labetalol, and propranolol were observed (Figure 1). Considering that carvedilol and labetalol have an additional α1-blocker mechanism, and Figure 2 highlights the superior efficacy of R-carvedilol, the question arises as to whether the main anti-tumor mechanism is due to α receptor blocking. We were able to confirm that several UM cell lines expressed α- as well as ß-adrenoreceptors (Figure 7). However, studies on skin carcinogenesis have suggested that the observed anti-tumor effects of carvedilol are independent of adrenergic receptor blocking, although the exact mechanism remains unknown [39]. In other studies, carvedilol has exhibited anti-oxidative and anti-proliferative activities and inhibited the PI3K/AKT and cAMP/CREB signaling pathways [17,40,41,42]. Thus, the anti-tumor effects observed in our study may be due to the inhibition of multiple oncogenic mechanisms and need to be explored in further studies.

The therapeutically administered dose of carvedilol as an anti-hypertensive drug is commonly 25–50 mg daily, divided into two intakes, which leads to limitations in the comparability of the concentration used in our study. We demonstrated the anti-tumor activity of carvedilol at concentrations of 10–50 µM in spheroid and cell culture experiments, whereas a single dose of 25–50 mg in patients has been found to result in a maximal plasma peak concentration of <1 µM [43,44,45]. Nevertheless, long-term use of carvedilol as an anti-hypertensive drug in a daily recommended dose was associated with a reduced risk of many cancer types in a recent population-based cohort study, suggesting carvedilol as a preventive tumor agent [46]. Another retrospective cross-sectional study of breast cancer patients revealed that non-selective ß-blockers, including carvedilol, reduced the tumor proliferation of early-stage breast cancer. In that same study, the prospective approach described the reduction in the breast cancer proliferation index by comparing pre- and post-propranolol intake. [47] Along with these direct anti-tumor effects of ß-blockers, adrenergic stress reduction and hypertension themselves have been shown to increase the risk of cancer and metastasis [48,49]. However, it is currently unknown whether stress or hypertension increase the risk of metastatic UM. If so, patients with UM and hypertension might benefit from switching to anti-hypertensive drugs with additional anti-tumor effects, such as carvedilol or propranolol. In contrast, the non-ß-blocking R-carvedilol could be used as a supplement adjunct to existing anti-tumor therapies such as radiation, resection, or enucleation, to minimize adverse cardiovascular effects. However, R-carvedilol is currently not clinically available, whereas carvedilol is an FDA-approved, worldwide available and affordable drug that could be administered to UM patients immediately [50]. Topical administration could enhance bioavailability during tumor treatment while minimizing adverse cardiovascular effects. Transdermal delivery systems based on nano lipid transferosomes incorporated into gels or patches have already been developed for the therapy of skin cancer [51,52]. In mice exposed to chronic UV radiation, topical carvedilol gel at 10–100 µM significantly delayed the incidence of skin tumors and reduced tumor number and burden with negligible systemic effects [52,53]. Interestingly, topical treatment with 10 µM R-carvedilol, but not racemic carvedilol, delayed tumor formation in a chronic-induced skin cancer mouse model [54]. Similarly, topical treatment, such as episcleral patches loaded with carvedilol or R-carvedilol, could be used to treat uveal melanoma.

Moreover, 3D culture systems can simulate tumor growth characteristics in vitro. In addition, the limited drug penetration of spheroids leads to a better comparability of tumor drug use [27,28]. The spheroids were treated with ß-blockers in their culture wells to preserve a functional tumor environment, resulting in representative results. The different 3D tumor spheroids of Mel270, 92-1, UPMD2, and UPMM3 cell lines may reflect the heterogeneity of UM. The different responses of the tested spheroid types to the treatment could be attributed to differences in intrinsic tumor cell responsiveness, as well as to distinct dimensions or compactness. The most responsive cell line, UPMM3, was obtained from a tumor with a monosomy 3 gene risk profile; these tumors are associated with a high metastasis rate, and thus a worse outcome [8,29]. However, consistent with our earlier findings, UPMM3 had a long doubling time and aggregated into smaller spheroids when compared to the other investigated spheroids [32]. To allow a better comparison of carvedilol sensitivity to each cell line, we additionally conducted experiments with 2D monolayer cultures. These results confirmed the high sensitivity of the monosomy 3-containing UPMM3 cell line and all other UM cell lines to the drug. However, in the larger and more compact spheroids of the cell line 92-1, a higher dose of carvedilol was required to block viability (Figure 6). The differences in viability observed among the different spheroids were most likely due to the lower penetration of carvedilol into the more compact 92-1 spheroids. When translated to the clinic, this would imply that larger tumors require higher doses of carvedilol to elicit substantial anti-tumor responses, irrespective of the underlying genetic risk status. Further studies are needed to confirm the anti-tumor effects of carvedilol and its R-enantiomer in vivo and to rule out adverse effects of higher doses on healthy ocular tissues or cells.

Since Episcleral plaque brachytherapy is one of the most effective treatments for UM, another aim of this study was to investigate the feasibility of combining carvedilol with UM radiotherapy. For this purpose, the spheroids derived from the UPMM3 cell line were additionally irradiated with 5 or 10 Gy. The results indicated that carvedilol can be co-administered with radiation without compromising the effectiveness of either carvedilol or radiation. Carvedilol is known to have anti-oxidant pharmacological properties and may protect against free radicals, which could interrupt radiation-induced damage to UM [55,56]. In contrast, our study suggests that carvedilol combined with radiation can block the repopulation of tumor cells more efficiently than radiation on its own (Figure 8). This combination treatment could limit side effects while preventing the long-term risk for tumor recurrence or metastasis. Collectively, the present preclinical study demonstrates for the first time the anti-tumor potential of carvedilol in UM. Further studies are warranted to evaluate carvedilol as an adjunctive pharmacological therapy for UM.

## 5. Conclusions

Our study has demonstrated that a specific group of non-selective ß-blockers exhibit anti-tumor potential for UM. Carvedilol and its non-ß-blocking R-enantiomer were the most potent elicitors of anti-tumor responses in UM spheroids. These responses included a reduction in viability and the induction of apoptosis and necrosis. However, in vivo studies such as chick chorioallantoic membrane or mouse models are warranted and planned to determine the effect of carvedilol or R-carvedilol on healthy tissue. Nevertheless, the results suggest that combined treatment with carvedilol and tumor irradiation could be used to treat radioresistant tumors and to ensure tumor control, setting a starting point for new additive therapeutic approaches in the treatment of uveal melanoma.

## Figures and Tables

**Table 1:** Characteristics of uveal melanoma cell lines.

Cell Line	Cytogenetics	Cell Morphology/Doubling Time	References
92-1	GNAQ Q209L, disomy 3, EIF1AX mutant	Epithelioid/38–58 h	[7,30]
Mel270	GNAQ Q209P, disomy 3	Spindle/43 h	[7,31]
UPMD2	GNA11 Q209L, disomy 3	Epithelioid/150 h	[29]
UPMM3	GNAQ Q209P, monosomy 3, BAP1 mutant	Spindle and epithelioid/100 h	[29]

## Data Availability

The data presented in this study are available in the article.
